# Can NLR, PLR, and LMR be used as prognostic indicators in patients with pulmonary embolism? Author’s reply on commentary

**DOI:** 10.17305/bjbms.2020.5292

**Published:** 2021-08

**Authors:** Nuri Köse, Tarık Yıldırım, Fatih Akın, Seda Elçim Yıldırım, İbrahim Altun

**Affiliations:** 1 Department of Cardiology, Private Mugla Yucelen Hospital, Mugla, Turkey; 2 Department of Cardiology, Faculty of Medicine, Balikesir University, Balıkesir, Turkey; 3 Department of Cardiology, Faculty of Medicine, Mugla Sitki Kocman University, Mugla, Turkey

We appreciate the comments made by Dr. Bedel *et al*. NLR, PLR, and LMR are affected by various diseases such as oncological, collagen tissue, inflammatory, or severe renal/liver diseases [1]. Due to this, we have listed some of the disorders mentioned above in the tables. Hematological diseases, collagen tissue disease, inflammatory diseases, congenital heart disease, or severe renal/liver disease were excluded from the study. However, the presence of malignancy did not affect our results in the regression analysis.

Platelets swell until 120 minutes in ethylenediaminetetraacetic (EDTA) and until 60 minutes in citrate [2]. The authors suggest that optimal measuring time should not exceed 120 minutes. The blood samples of the patients were taken within 1 hour after their emergency admission. All blood samples in our study were tested within 1 hour of collection [3]. We used EDTA for whole blood anticoagulation. The mean duration of symptoms before admission was 5.04 ± 6.9 days.

The drugs such as corticosteroids affect inflammatory parameters. Therefore, we excluded inflammatory diseases without emphasizing corticosteroids or other anti-inflammatory drugs.
